# Uganda at Glance of 5.7 Magnitude Earthquake: Lessons for Earthquake Risk Reduction

**DOI:** 10.1371/currents.dis.646967e849bc40bfb5d9cd54b66a2eee

**Published:** 2018-10-30

**Authors:** Joseph Kimuli Balikuddembe, Paul Sinclair

**Affiliations:** Institute for Disaster Management and Reconstruction, Sichuan University, Chengdu - China and Hong Kong Polytechnic University, China.; One World One People, 4 Poskitt House, Waverley Road, Bridgewater, TA63RZ, UK

## Abstract

Introduction: Uganda remains seismically vulnerable to earthquakes, which constitute one of the most deadly naturally triggered disasters in the world. This is not surprising given the country’s location in the East African Rift Valley System.

Method: This paper draws mainly on the authors’ live event experience and some media reports to narratively outline the nature of a sizable earthquake, which measured a magnitude of 5.7 on the Richter scale that struck Uganda and other countries within the Lake Victoria Basin region on 10th September 2016 in the afternoon.

Results: Rakai - a district in central region was the worst affected in Uganda. It witnessed the death of four people; 20 people were admitted to the hospital with injuries; a total of 590 people were affected; and serious structural damages mainly in buildings were reported, leaving many either razed to the ground or left with cracks.

Discussions: Although this earthquake was less devastating in terms of injuries and fatalities compared to two previous earthquakes in Uganda, based on the Modified Mercalli Intensity Scale it was still considered to be severe. Therefore, this paper identified some proactive lessons as far as earthquake risk reduction in Uganda is concerned, which among others include: encouraging earthquake-resistant buildings; the safety of essential infrastructure; earthquake early warning systems supported by free global technologies; and the safety of rescue workers along with prioritizing the psychosocial needs of rescue teams. With all this in mind, the September 2016 earthquake should serve as a timely reminder that there is a real need for the proactive ex-ante earthquake preparedness rather than risking an expensive post-ante approach to responding to any future devastating earthquakes in Uganda.

Keywords: Earthquake, Uganda, disaster risk reduction, ex-ante approach, post-ante approach

## Introduction

Earthquakes are spontaneous events that constitute one of the most deadly naturally triggered disasters, which can cause extensive fatalities, injuries, disabilities, displacement, physiological distress, extensive property damage and devastating economic losses^1^^,^^2^^,^^3^. Earthquakes are a result of sudden energy releases in the earth’s crust, which create seismic waves that result in the ground shaking^4^. They are often followed by a series of aftershocks or violent shakes, which can have a significant impact and do damage especially to already weakened buildings and other essential physical infrastructure. As inhabitants of a seismically active planet^5^^,^^6^**, **large percentages of the populations in different countries are predisposed to earthquakes of varying magnitudes and intensities. The countries situated in the seismically active areas, however, are far more prone to the earthquakes and their associated impacts.

Earthquakes are known to trigger geohazards such as landslides, mudslides, tsunamis, avalanches, floods, volcanic eruptions, fires, technological accidents, water contamination, epidemics and panic-driven stampedes in crowded areas^7^^,^^8^. Other geohazards are linked to catastrophic soft-sediment deformation which can lead to liquefaction and fluidization of near surface sediments^9^. Some geohazards may take weeks, months or even years before they manifest. Historically, catastrophic earthquakes of different magnitudes (M) have occurred all over the world. The most destructive earthquakes in terms of fatalities and damage ever witnessed in the past decade were: the 6.9M Gujarat - India 2001; 6M Bam - Iran 2003; 7.6M Kashmir – Pakistan 2005; 7.9M Ica-Peru 2007; 8.0M Sichuan - China 2008; 7.2M Port-au-Prince - Haiti 2010; 9.0M Tohoku-Oki – Japan 2011 and 7.9M Nepal 2015^3^^,^^4^^,^^6^^,^^10^^,^^11^^,^^12^. These events left widespread devastation with death tolls in hundreds of thousands, wide scale injuries and disabilities. They also left many survivors with short-term and long-term physiological and mental problems which emanated mostly from the socioeconomic stress. Added to their problems were the devastating property losses and widespread damage to essential infrastructure that often became inoperable.

The Lake Victoria Basin, which includes Uganda, remains a seismically active area and it has experienced many earthquakes including the most recent one which occurred on 10th September 2016. Since Uganda is one of the countries located in the seismically active areas, what was the nature of the September earthquake? Also, what lessons are to be derived as far as earthquake risk reduction in Uganda is concerned?

## Method

This paper draws mainly on the authors’ live event experience and reviews of some media reports to narratively outline the nature of a sizable earthquake which measured a magnitude of 5.7 on the Richter scale that struck Uganda and other nearby countries within the Lake Victoria Basin region on 10th September 2016 in the afternoon. After explaining its nature, the present paper identifies and divulges some proactive lessons as far as earthquake risk reduction in Uganda is concerned.

**Description of the September 10th 2016,** **5.7 Magnitude Earthquake in the East Africa**

On 10th September 2016 (Saturday) at 15:27:28 local time (12:27:28 GMT/UTC Time), the Lake Victoria Basin region experienced an earthquake measuring a magnitude of 5.7 on the Richter scale^13^. The earthquake’s epicenter was located east of the north-western town of Nsunga, - Kagera region in Tanzania on the border of Lake Victoria and it triggered a green Global Disaster Alert Coordination System (GDACS)^14^. The earthquake tremor shocks were felt for 20 seconds to 3 minutes across different parts of Uganda, and rippled out as far as Rwanda and western parts of Kenya – which are all parts of the Victoria Basin. Its depth was reported to be only 23 kilometers (~15 miles) and this was considerably shallow. On the Modified Mercalli Intensity (MMI) Scale 1, the earthquake measured 7.99, which can either be considered very strong or severe.

It should be noted that the four countries which experienced this earthquake are situated in the East African Rift Valley System (EARVS), which is known to have significant seismic fault lines running through it^15^. The EARVS is formed by a 3,000-km-long Cenozoic age continental rift extending from the Afar triple junction (between the horn of Africa and the Middle East) Mozambique in the West. Preliminary information indicated that the quake was a result of shallow oblique faulting within the lithosphere of the African plate. This caused a rupture on a moderately dipping fault line striking either the Northeast-Southwest (right-lateral slip) or East-West (left-lateral slip) or east-west (left-lateral slip)^14^. Following the earthquake’s main-shock, there were lighter aftershocks reported the next day (Sunday 12th September, 2016) in the affected areas in Tanzania that had been struck by the earthquake

According to the United States Geological Survey (USGS), the origin of the earthquake was some 200 km or more from the east and west branches of the EARVS^14^**. **The EARVS which extends from the Red Sea/Gulf of Aden to Malawi is formed by the western and eastern rift branches^16^. The eastern branch runs North-to-South from Afar Depression in Ethiopia through Kenya and central Tanzania, several hundred kilometers from the origin of the earthquake. The western branch runs from Southern Sudan to Western Uganda, Rwanda, Burundi and Tanzania along the boundary with the Democratic Republic of Congo^14^^,^^17^. So the Victoria Basin lies between the two rift branches, and is susceptible to their divergent tectonics whenever the rift segments are connected and cause divergent strike-slip motions. Earthquakes occur as a result of tectonic plates colliding and from the sudden energy releases in the earth’s crust, which create seismic waves that result in the ground shaking^4^. This is what happened to trigger the 2016 September earthquake on the Victoria microplate, in an area that had little to no recorded earthquakes over the past century^14^**.**

**Earthquakes and Seismological Monitoring System in Uganda**

Records of Uganda’s vulnerability to earthquakes date back to 1897^18^**.** Over the years, earthquake shocks of varying magnitudes have been recorded in different parts of the country – especially in the Rwenzori Mountains which is one of Uganda’s most seismically active areas. The Fort-Portal area, which is proximate to the Rwenzori Mountains, recorded 418 shocks out of all the 588 shocks that were felt in Uganda as a whole between 1907 and 1942. Other places that have seen significant numbers of shocks between 1910 and 1960 include Mubende (55 shocks), Mbarara (46), Hoima (36), Entebbe (30), Kabale (23) and Kampala (6). These areas are known epicenters of tremors in Uganda^8^**. **Tremors vary in magnitude but the most destructive earthquakes so far in the history of Uganda, occurred on the 20th March 1966 in the Toro region and on the 5th February 1994 in the Kabarole district measuring 6.6 M and 6.2 M respectively. In the Toro region earthquake, 150 people were killed and 1,300 injured while in the Kabarole district earthquake 8 people were reported to have been killed together with property destruction estimated at US$ 61 million. The two events were linked to fault rifting in the EARVS^8^^,^^19^**. **Other areas of seismological importance include the Rwenzori mountains which recorded approximately 800 seismic events per month with local magnitude ranging from -0.5 to 5.1 between February 2006 and September 2007^15^. Aside from that, the reported 2016 earthquake is another noteworthy earthquake that affected Uganda and it is worth discussing.

In response to the geological risks, Uganda established a seismological monitoring system which is monitored through the National Seismological Network (NSN). Potential earthquakes and their related hazards in Uganda are monitored from four seismic stations which form the NSN, under the Department of Geological Survey and Mines. The stations are located in Entebbe, Hoima, Kilembe and Mbarara. They use the station codes of ENTT, HOI, KIL and MBAR respectively^19^^,^^20^^,^^21^**. **ENTT, HOI and KIL use MEQ-800 analogue ORION Digital equipment while MBAR uses special equipment as part of the Global Seismological Station Network (IRIS-GSN). The equipment automatically collects, transmits, processes, analyzes and interprets real-time waveform data using Seisan Earthquake Analysis Software^20^. In turn, this helps to produce updated information for the national earthquake database. That can then be used to generate seismicity hazard maps; monitor the seismicity impacts which are likely to be caused by the ongoing activities in the country and to provide seismic data that can be shared amongst the partners, neighboring countries and other parties^20^^,^^21^**. **Ultimately, this should help to inform the timely detection, preparedness and response to earthquakes and their related hazards.

The seismological monitoring would, however, be much more effective if it was utilized to create a well-organized and comprehensive earthquake damage risk reduction policy. Unfortunately, at present Uganda does not such a policy. Instead, some few earthquake preparedness actions and codes are included in the National Policy for Disaster Preparedness and Management (NPDPM) and the Seismic Code of Practice for Structural Designs (SCPSD) respectively^22^^,^^23^**.** The NPDPM articulates the measures which are related to mapping earthquake prone areas; developing public awareness on earthquake preparedness; developing earthquake resistant building standards and ensuring adherence to building codes and regulations. Other measures specified within NPDPM include: conducting geological studies and research; promoting seismic safety activities and acquiring the relevant technology to monitor and detect the occurrence of earthquakes. The Ministry of Energy and Mineral Development is designated as the leading authority in the field of earthquakes and it collaborates with other stakeholders to implement these measures^22^. All this notwithstanding, much more is still needed to be done. This is why there is an urgent need for an earthquake risk reduction policy to not only support the timely detection, transmission and processing of data pertaining to the occurrence of any potential earthquakes but also to co-ordinate effective emergency response actions to deal with their potentially catastrophic impacts in Uganda.

**Uganda’s Experience with the September 2016 Earthquake**

This earthquake caught the vast majority of the people by surprise, despite the presence of seismological monitoring systems in Uganda as noted above, which should detect any potential earthquake occurrence in a timely manner^20^^,^^21^^,^^22^^,^^24^. This earthquake happened in a weekend and in the afternoon when many people were in their homes, or visiting shopping malls and places used for leisure and entertainment. Others were in offices and at work places. The quake was experienced as a strong vibration that rattled the buildings people were occupying. Some people were frightened to see furniture, doors and windows violently shaking and rumbling, cabinets opening, glass panes warping and weak hanging items falling from the walls. Others mistook it for a bomb blast. As a result, many panic-stricken people ran and scampered to exit buildings for their safety^13^^,^^25^. The earthquake almost shook the whole country at varying time intervals, but the worst shaking was felt in the central and the southern parts of Uganda.

While Uganda felt the consequences of the earthquake, Tanzania, a neighbor of Uganda in the South was much more affected by it. Tanzania’s Kagera region was reported to have been the most devastated with ~11 deaths, ~440 injuries and the total estimated economic damage at $ 458 million^13^^,^^14^. Rakai - a district located in central region was the most affected area in Uganda **(figure 1)**. This is because the district stretches to the Uganda-Tanzanian border, close to the Kagera region which was the epicenter of the earthquake. Only four people were reported dead, and a total of 590 people were affected mainly in Rakai district. Based on this, the fatality rate was low if compared with more or less similar earthquakes of the same magnitude in Uganda and elsewhere for example the 1994 Kabarole 6.2 earthquake and 2003 6M Bam in Iran. Apart from the 20 people who were admitted as patients to the Kakuuto hospital with serious injuries, it was further reported that a local medical nurse at Kateera Health Center II sustained head injuries.

Given that the need to make vital infrastructural buildings earthquake resistant cannot be over emphasized– especially key emergency medical and health buildings. A similar situation, though with far more devastating consequences to health facilities and workers, was witnessed in the major earthquakes of Bam, Gujarat, Haiti, Ica, Kashmir, Sichuan and Tohoku-Oki^3^^,^^4^^,^^10^^,^^11^^,^^27^. It was reported, for example, in the Bam earthquake that, all the health facilities in the affected areas were destroyed, with a loss of almost 50% of local health staff. Essential physicians and nurses who could not easily be replaced were also killed and injured^10^.

Earthquakes are known to cause immense economic losses, which occur either immediately or after long-term periods^4^^,^^5^^,^^7^^,^^28^. In regard to the September earthquake in Uganda, its immediate economic losses were experienced soon after it had occurred in terms of structural and non-structural damages. This was mostly felt by lower income households in the Rakai district. Accordingly, it was reported that more than 500 houses and a police post collapsed and were razed to the ground. Another 3,186 houses were left with cracks from the earthquake tremors^25^**. **As a direct consequence, the families which were most affected became homeless since they had to abandon their houses to sleep in plantations**.** Others were left with little or no choice other than to continue sleeping in their quake-damaged houses. However, there has been no clear estimates to date of the real monetary value of the losses and damages that were calculated or projected to be associated with the earthquake unlike in Tanzania as noted above which recorded $ 458 million^26^.

**Map and location of Rakai district in Uganda (Source: NPA,2011).**
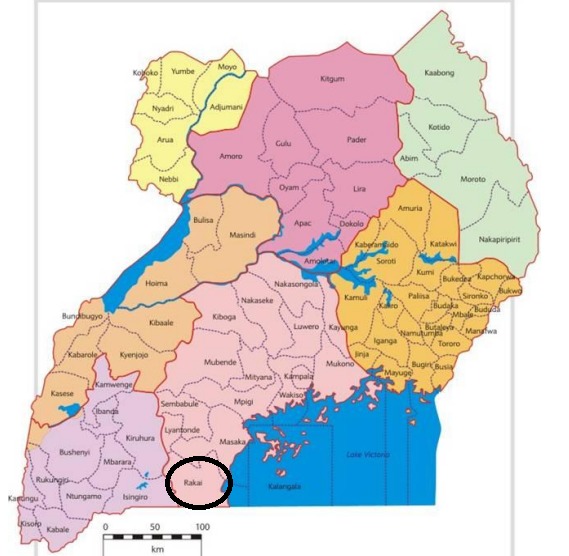

Shallow earthquakes generally tend to be more damaging than deeper quakes whose seismic waves have to travel farther to the surface. The farther they travel the more kinetic energy they lose. This is why the shaking caused by shallow quakes may cause large seismic slips, which can eventually lead to mega-thrust earthquakes^11^. Uganda and other E.A countries, which experienced the earthquake, should therefore consider themselves somewhat fortunate that the depth was reported to be at 10 km only. At this depth the quake was far less destructive when compared to the devastating earthquakes already cited above - the Indian-Gujarat, Iranian-Bam, Pakistan-Kashmir, Chinese-Sichuan, Haitian-Port-au-Prince, Japanese-Tohoku and Nepal. Most of the earthquakes required emergency response actions that were based on the management of mass casualty incidents, which was not the case with this earthquake that affected Uganda and other E.A countries.

However, emergency shelter (tents) and basic facilitates were much needed for the homeless or displaced victims whose homes were made unsafe to inhabit and accommodate them. This paper argues that the affected people would have been unnecessarily exposed to disease outbreak such as malaria, diarrhea and acute respiratory infections. Many were left in situations that could have exacerbated their vulnerability to psychosocial distresses like posttraumatic stress disorders. Additional injuries from the falling materials inside the cracked houses were inevitable to those who continued to reside in the quake damaged homes which were then further damaged by aftershocks. More so, the problems for those most affected by the earthquake did not end there. Some victims might have been expected to pay for the costs of the transportation and the treatment of the injured as well as for the repair of damaged structures. This situation not only exposed victims to health-related risks but it also exposed them to further impoverishment at a time when their already meager incomes were needed for providing for their family’s most basic needs.

**Is Uganda Prepared to Respond to Potential Earthquakes and Related Hazards Based on the Nature of the September 2016 Earthquake?**

There is no escaping the fact that Uganda will continue to be vulnerable to future earthquakes given its location in the EARVS^8^^,^^15^^,^^17^^,^^21^**. **This situation is also heavily compounded by the ongoing political and socioeconomic dynamics in the country. One of the noteworthy challenges is the inappropriate construction methods and materials often used for different types of building structures in Uganda. They fail to comply with the existing building engineering codes to make them structurally more stable and resistant to earthquakes. Buildings which fail to comply with required construction codes can easily collapse on their occupants during earthquakes. It is important to note that, the number of buildings being built in many urban areas of Uganda is skyrocketing but unfortunately many of them are non-compliant with the essential building codes. This problem has been of particular concern in Kampala, the capital city, which lies in a seismically active zone next to Lake Victoria and hosts a population estimated at 4.5 million. Only recently has more stringent construction measures started to be enforced.

Uganda as a whole is vulnerable to incurring heavy fatalities from earthquakes in places of excessive population growth. Mostly the riskiest places are those which are characterized by dense and informal settlements. Kampala is a case example with many earthquake susceptible housing structures which are compounded by the unregulated development of 62 informal settlements (slums)^29^^,^^30^. Of particular concern are the adobe-brick constructed houses that have been increasingly built upwards to accommodate the increasing numbers of poverty strickened populations. In fact, the adobe-brick housing structures have been increasing at 60% per annum compared to past years^31^. Makeshift structures made of mud and wattle that are common especially in rural and remote areas like Rakai are also particularly vulnerable to collapsing during earthquakes. Therefore, the recent earthquake should be seen as a wake-up call for the need to come up with ways to strengthen even low-cost basic structures so that they will be much less likely to collapse on their inhabitants during earthquakes.

There is also a great need to enforce building codes and to ensure where possible that quality earthquake-resistant building designs are implemented. Needless to say, that is absolutely essential for vital infrastructural buildings including those that are essential for the delivery of vital services such as the police stations (like the one that was destroyed by the earthquake in Rakai district). The buildings of that nature need to comply with the minimum standards of the existing SCPSD regulations. Also, where possible the SCPSD should be regularly revised to address the changing construction and engineering dynamics whenever any important lessons are learned from both naturally triggered and human-induced disasters around the world. It’s quite evident that new technology and improved construction methods and materials continue to be developed. So it’s important that the SCPSD keeps pace with these changes so that any of the important opportunities they advance to make cost-effective improvements are leveraged.

Besides the case with protecting hospitals and police stations as noted above, it’s also evident that earthquakes often damage and destroy essential infrastructure such as roads, airports, telecommunication facilities and networks, electricity and water infrastructure^5^^,^^6^^,^^7^^,^^10^. These facilities are often vital to coordinating the rapid needs assessment and delivery of emergency relief assistance to victims. Immediately after the occurrence of the earthquake, some relief aid was delivered to the victims and along with that the Ugandan Government promised the affected households building materials such as cement, iron sheets and iron rods to help victims repair and rebuild their houses^32^. Though these efforts are commendable much more needed to be done to ensure that this aid material was used most effectively. The coordination and sustainability of relief efforts also depends on vital infrastructure. Weak and inaccessible roads; inadequate social services, poor telecommunications and high rates of poverty can hamper any timely relief interventions as happened to the victims in Rakai district. The lessons that can be learned from that are particularly evident when we consider some of the factors that were noted to have hindered the relief coordination among the non-governmental-organizations and the Haitian Government during the response to the 2010 Haitian earthquake^12^**. **This paper notes that the September earthquake in Uganda should be an opportunity for enhancing the resilience and safety of essential infrastructure, along with the need for preplanning emergency responses.

Earthquake early warning systems (EEWS) can help to greatly minimize the consequences of earthquakes by providing better understanding of their precursory activities^7^. It should be noted that prior to the occurrence of the recent earthquake there was no official warning information notifying the populations at most risk or the general population in Uganda, despite the seismological monitoring systems in the country which are in place to detect any unusual seismic activities. Therefore, the recent earthquake should be seen as an opportunity for Uganda to strengthen and improve its seismological monitoring systems, and other disaster early warning systems (EWS) to facilitate timely hazard detection, preparedness and response. This however ought to be aligned with multidisciplinary support and collaboration from both local and international stakeholders. It should also encourage greater use of affordable or free global technologies such as the Prompt Assessment of Global Earthquakes for Response (PAGER) and GDACS. Ideally, these can help in comparing their data with that generated by the existing seismological monitoring systems so that any deficiencies can be quickly identified and remedied. Also, community engagement coupled with public awareness campaigns to educate people about what to do in the event of an earthquake are highly required. Further research should also be undertaken so as much can be learned as possible from the previous earthquakes which either struck or affected Uganda including this recent one. Japan in particular offers a lesson to Uganda even though the two countries are at a different level of development. Between 2003 and 2007, Japan installed the most advanced earthquake and tsunami EWS and that considerably helped to save countless lives and reduced the losses during the 2011 Tohoku earthquake^27^.

Aside from caring for traumatized victims, the psychosocial needs of the rescue teams are an important driver to be prioritized to enable the success of the response activities to disasters. At times, some response measures undertaken by the rescue workers during disasters like earthquakes can be complex and expose them to life changing and potentially deadly occupational hazards. For instance, responding effectively to the needs of victims after the occurrence of earthquakes by providing the emergency medical care to any severely injured people; searching for and rescuing the victims entrapped in the wreckage. Similar attention needs to be prioritized for the firefighting and rescue workers who at times find themselves at high risk of losing their own lives or developing physiological distress^33^^,^^34^. This emanates mostly from encountering the deaths of victims who could not be saved; supervisory work overload; lack of rest; lack of vital communication; the death or loss of colleagues who go missing and near-death experiences among other factors^34^. With the case of this earthquake as noted above, one local medical nurse suffered serious head injuries. This is a lesson for Uganda that major disasters other than this earthquake can pose serious threats to the rescue teams especially if they are not trained or prepared properly, or they are poorly equipped or they are not supported adequately during disaster events. Therefore, there’s much that has to be done to ensure adequate safety of rescue workers as well as to maintain their morale high in times of responding to distressful disasters and emergency situations.

## A Call for Adequate Preparedness to Earthquakes

This paper aimed at outlining the nature of the 2016 September earthquake as well as identifying some lessons for earthquake risk reduction in Uganda. Besides, some of the lessons could also be applied in responding to other disasters and emergency situations in Uganda in general. With the above review in mind, the 2016 September earthquake should serve as a timely reminder that there is a real need for the proactive ex-ante earthquake preparedness rather than an expensive post-ante approach to responding to future earthquakes. This applies to not only the Rakai district and other known earthquake prone areas, but to Uganda as a whole as far as earthquake risk reduction in Uganda is concerned. However, that needs to be aligned to the SCPSD and some policy actions enshrined in the NPDPM, and above all the Sendai Framework for Disaster Risk Reduction 2015 – 2030 with special attention given to its priority number four: enhancing preparedness for effective response and to “Build Back Better” in recovery, rehabilitation and reconstruction.

## Data availability

All relevant data are indicated within this paper, and it does not report new data apart from that herein referenced.

## Funding

This paper received no funding.

## Competing Interests

The authors have declared that no competing interests exist.

## Corresponding Author

Joseph Kimuli Balikuddembe, Email: jbalikuddembe.k@scu.edu.cn
